# Mechanotransductive stabilization of HIF-1α is inhibited by mitochondrial antioxidant therapy in the setting of pulmonary overcirculation

**DOI:** 10.1038/s41598-025-99062-0

**Published:** 2025-05-10

**Authors:** Jason T. Boehme, Sanjeev A. Datar, Xutong Sun, Wenhui Gong, Qing Lu, Jamie Soto, Michael A. Smith, Alejandro E. Garcia-Flores, Gary W. Raff, Ting Wang, Emin Maltepe, Stephen M. Black, Jeffrey R. Fineman

**Affiliations:** 1 Department of Pediatrics, University of California San Francisco, 513 Parnassus Ave., HSE 1401 Box 1346, San Francisco, CA 94143-2205 USA; 2 Center for Translational Science, Florida International University, 11350 SW Village Parkway, Port St. Lucie, FL 34987-2352 USA; 3 Department of Environmental Health Sciences, Robert Stempel College of Public Health and Social Work, Florida International University, Miami, FL 33199 USA; 4 Department of Surgery, University of California Davis, Davis, CA 95817 USA; 5 Cardiovascular Research Institute, University of California San Francisco, San Francisco, CA 94143 USA; 6 Department of Cellular Biology and Pharmacology, Howard Wertheim College of Medicine, Florida International University, Miami, FL 33199 USA

**Keywords:** HIF-1α, Mitochondrial ROS, Mechanotransduction, Nitric oxide, Pulmonary hypertension, Congenital heart disease, Vascular diseases, Cardiovascular diseases

## Abstract

In patients with congenital heart disease, the development of pulmonary arterial hypertension (PAH) is based on vascular exposure to abnormal hemodynamic forces. In our work using a large animal model of increased pulmonary blood flow and pressure, we have previously described a pattern of alterations to vascular cell metabolism, mitochondrial function, and mitochondrial redox signaling, paralleling changes in advanced pulmonary vasculopathy states. Based on our findings and emerging literature, we believe that endothelial mitochondria play a central role in integrating and relaying pathologic mechanotransductive signals in abnormal pulmonary hemodynamics. In this manuscript, we demonstrate that exposure of the pulmonary vascular endothelium to aberrant mechanical forces increases production of mitochondrial reactive oxygen species (ROS) and stabilizes the transcription factor Hypoxia Inducible Factor-1α (HIF-1α), and that these changes are associated with impaired endothelial production of Nitric Oxide (NO). We validate that the mitochondrial antioxidant 10-(6′-ubiquinonyl)decyltriphenylphosphonium bromide (MitoQ) can reverse these alterations in vitro, and evaluate the effects of MitoQ treatment in vivo utilizing our large animal shunt model. We find that MitoQ therapy in pulmonary overcirculation decreases the production of mitochondrial ROS, diminishes the mechanically-induced stabilization of HIF-1α, and partially restores vascular reactivity by rescuing endothelial NO production. These findings raise exciting prospects concerning shared pathophysiologic mechanisms and possible common therapeutic targets amongst PAH etiologies.

## Introduction

Pulmonary arterial hypertension (PAH) is a complex phenotype that develops in response to a wide range of inciting pathologic stimuli, with advanced disease states characterized by progressive vasculopathy and proliferative remodeling of the pulmonary arteries (PAs). Despite the diverse range of underlying clinical risk factors, the cellular mechanisms underlying this proliferative vasculopathy exhibit notable commonalities and recurring themes. These include alterations towards pro-proliferative and anti-apoptotic growth, fibrosis and changes in the extracellular matrix composition, abnormalities of growth factor signaling pathways, particularly of the TGFβ and BMP superfamily, vascular inflammation and aberrant immune cell interactions, along with alterations in mitochondrial function, metabolism, and redox signaling^1–3^.

In patients who develop PAH associated with congenital heart disease (CHD), the major risk factors that dictate the early progression of disease are hemodynamic. Increased pulmonary blood flow seems necessary, though lesions that expose the pulmonary vasculature to high pressures exhibit the most significant risk and fastest progression of vasculopathy.^4^ Our work leverages a large animal model of increased pulmonary blood flow and pressure to characterize the mechanotransductive pathways that detect and integrate these aberrant mechanical forces into pathologic cellular signaling cascades.^5^ Utilizing this clinically relevant model, we have identified a pattern of significant alterations to cellular metabolism, mitochondrial dynamics and function, and mitochondrial redox signaling, involving both the arterial and lymphatic vascular beds.^6–9^ These findings parallel the changes described in more advanced states of pulmonary vasculopathy^2^,^3^ and exhibit complex interrelationships with vascular function and cellular growth phenotypes, even at the early stage of vascular disease captured by our model. ^6–9^.

Our observations are consistent with an increasing body of literature that identifies mitochondria as mechanoresponsive organelles that play an important role as mediators of mechanotransduction. Mitochondria adapt to cellular stresses and fluctuations in energy demand physiologically and through dynamic alterations in size, shape, and intracellular distribution.^10^ These morphologic alterations include translocation, elongation and cristae remodeling, along with mitochondrial fission and fusion, all of which are shown to be deeply interconnected with mitochondrial function and metabolism.^10^ Mitochondrial morphodynamics have been demonstrated across multiple cell types to react to diverse mechanical stimuli, including stiffness of the extracellular matrix or substrate environment,^11,12^ cellular stretch,^13^ shear forces,^14^ and hydrostatic pressure.^15,16^ Elegant work by several groups has demonstrated that these mechanoresponsive changes in morphodynamics coincide with alterations in metabolic activity and, importantly, the production of mitochondrial reactive oxygen species (ROS).^12,17,18^.

Mitochondrial ROS are widely shown to play vital roles in physiologic and pathologic cellular redox signaling cascades, and are strongly associated with the development of pulmonary vascular disease.^2,19–21^ Based on our previous findings and observations from other groups, we believe that pulmonary endothelial mitochondria play a central role in integrating and relaying pathologic mechanotransductive signals in the setting of abnormal pulmonary hemodynamics. This signaling occurs, at least in part, through the modulation of mitochondrial ROS and the subsequent engagement of downstream redox-sensitive pathways, as exemplified by the normoxic stabilization of the transcription factor HIF-1α in our model.^7,8^ In this manuscript, we further evaluate this paradigm by assessing the in vitro and in vivo effects of the mitochondrial-targeted antioxidant mitoquinone (MitoQ) on pulmonary endothelial responses to abnormal hemodynamic forces.

## Materials and methods

### Lamb model, hemodynamic assessment and vasoreactivity testing

As previously described, late gestation fetal lambs from mixed-breed Western ewes underwent fetal surgical anastomosis of the left pulmonary artery and ascending aorta via an 8.0 mm GoreTex graft (shunt).^5^ Lambs were subsequently born spontaneously, and shunt animals in the MitoQ treatment group received daily enteral MitoQ at a dose of ~ 2 mg/kg rounded to the nearest 5 mg increment. Treated (N = 7) and untreated (N = 8) shunt lambs of mixed sex were followed to 4–6 weeks of age when they underwent hemodynamic assessment and tissue collection as described in detail previously.^22^ Briefly, animals were anesthetized with weight-based boluses followed by continuous infusions of fentanyl citrate, diazepam, and ketamine hydrochloride titrated to full relaxation and lack of response to painful stimuli. Neuromuscular blockade was administered with vecuronium bromide and maintained via continuous infusion. Under full sterile and aseptic surgical technique, fluid filled polyurethane catheters were placed via open thoracotomy in the main pulmonary artery (PA), femoral artery, and bilateral atria, and connected to pressure transducers. An ultrasonic flow probe (Transonic Systems) was placed on the left PA. Physiologic parameters were continuously recorded and analyzed using the Ponemah physiology platform (DSI). Animals were ventilated at an FiO2 of 0.21 and PEEP of 5 for hemodynamic assessment, and ventilatory parameters were adjusted for an arterial pH of 7.35–7.45 and PaCO_2_ of 35–45. A hemodynamic baseline was established and recorded following recovery from surgical procedures when sedation and blood gas parameters were achieved. Qp/Qs was calculated with simultaneous sampling of blood gases from the main PA distal to the surgical shunt, the right ventricle, the femoral artery and the left atrium to confirm an open and physiologically relevant shunt. Endothelial-dependent PA relaxation was assessed with rapid, direct main PA injections of Acetylcholine chloride (1 μg/kg) repeated in duplicate with at least 5 min for the animal to return to baseline between injections. After at least 10 min time allowed to return to hemodynamic baseline following Ach, endothelium-independent PA relaxation was assessed with administration of inhaled NO at 20 ppm (Inovent, Ohmeda) for 10 min. Finally, vasoconstrictive responses were assessed in response to hypoxia administered with a N_2_ bleed in and titration to FiO_2_ of 0.10 for a duration of 10 min. At the end of each study, lambs were euthanized with sodium pentobarbital followed by bilateral thoracotomy as described in the NIH Guidelines for the Care and Use of Laboratory Animals. Collection of tissues for biochemical assessment and pressure myography, and isolation of primary endothelial cell lines was performed. Animal experimentation was designed and conducted in accordance with ARRIVE guidelines. Given multiple prior comparisons of unoperated age-matched control animals, as well as preserved tissue and plasma samples and cell lines, contemporaneous unoperated controls were not repeated for these experiments. Animals were excluded from analysis only for Qp/Qs indicating lack of existing physiologic shunt at the time of procedure or major surgical complication that precluded performance of vasoreactivity testing. Data extraction of physiologic outcomes was performed by a single individual who was blinded to group. All protocols and procedures related to the care and evaluation of these animals were approved by the Institutional Animal Care and Use Committees (IACUC) of the University of California, San Francisco, and the University of California, Davis.

### Isolated pulmonary artery pressure myography

As described previously,^23^ 4th-5th generation PAs (inner diameters of ~ 1 mm) were dissected, isolated, and cut into rings for evaluation by wire pressure myography.^24^ Briefly, ring segments of PA were prepared and mounted on a multichannel myograph (DMT610M, Danish Myo Technology, Aarhus, Denmark) for dynamic measurements of isometric force development. 4 vessel segments per animal were mounted for recording. Vessels were maintained at 37 °C in physiologic saline solution (PSS, pH 7.4) equilibrated with a 21% O_2_ and 5% CO_2_ mixture throughout the experiments. Isometric force (in mN) was sampled at 20 Hz with a Powerlab8/30 (AD Instruments) using LabChart version 8 software. After mounting, ring segments were allowed to equilibrate for 45 min under zero tension in PSS with 21% O_2_ and 5% CO_2_. The ring segment response protocol included pre-constriction with NE to 80% maximum constriction (EC_80_), followed by evaluation of the relaxation response with titration of ACh (6 doses: 10^−9^–10^−4^ M). Only segments that developed peak active tension > 0.05 mN/mm were included for statistical analysis. Norepinephrine hydrochloride (NE) and acetylcholine chloride (Ach) were obtained from Sigma-Aldrich.

### Cell isolation and culture, shear, and stretch exposure

Primary cultures of pulmonary artery endothelial cells (PAECs) were isolated as described previously^9^ from explanted segments of the proximal pulmonary arterial branches. While multiple cell lines are derived from each animal, every cell line utilized for the experiments described in this manuscript was derived from a unique animal. Cells were maintained in Dulbecco’s Modified Eagle Medium with 1 g/L glucose, L-glutamine, and sodium pyruvate supplemented with 10% fetal bovine serum. All experiments were conducted in cells between passages 5 and 10. As previously described,^25^ laminar shear stress was applied using a cone-plate viscometer at 20 dyn/cm2 of shear stress for the indicated amount of time. Cyclic stretch experiments were performed as previously described.^26^ Briefly, PAECs were plated onto Bioflex collagen I type cell culture plates (FlexCell International, Hillsborough, NC) and stimulated for 4 h at 1 Hz and 18% amplitude (deviation from baseline dimension) on the FlexCell FX-5000 System.

### RNA sequencing and data analysis

Primary PAECs were isolated from the proximal pulmonary arteries of N = 3 untreated shunt animals and N = 4 age-matched controls and submitted for RNA sequencing analysis performed by Exiqon (Denmark) as previously reported.^27^ This previously published dataset was reanalyzed using QIAGEN Ingenuity Pathway Analysis (IPA).^28^ 15,808 distinct transcripts were mapped to the database, of which 1799 significant transcripts were identified based on differential expression between groups with a *p*-value < 0.05 corrected for multiple comparisons using the Benjamini–Hochberg procedure. Core and canonical pathway analyses were performed using a right-tailed Fisher’s Exact Test to determine the probability of association between the identified significant transcripts in the dataset matched against defined canonical pathways in the IPA knowledge base. A *p*-value < 0.05 was used as the threshold for significance.

### Determination of mitochondrial ROS in PAECs

Mitochondrial ROS were assessed as previously described^9^ by quantification of the mitochondrially-targeted superoxide probe MitoSOX Red (Molecular Probes, Grand Island, NY).^29^ MitoSOX was added to cells in culture media at a final concentration of 5 µM and allowed to rest for 30 min at 37 °C in the cell culture incubator, then imaged by fluorescence microscopy using an Olympus IX51 microscope equipped with a CCD camera (Hamamatsu Photonics). The average fluorescent intensities were quantified using ImagePro Plus version 5.0 software (Media Cybernetics).

### Measurement of superoxide levels in peripheral lung tissue by EPR

Electron paramagnetic resonance (EPR) measurements were performed using snap-frozen peripheral lung tissue from control, shunt, and MitoQ-treated shunt lambs as we have previously described utilizing the spin probe 1-hydroxy-3-methoxycarbonyl-2,2,5,5-tetramethylpyrrolidine (CMH).^22,30^.

### Measurement of bioavailable NO (NOx) from lung tissue, plasma, and cell culture media

Bioavailable NO (NOx) was measured using a Sievers 280i Nitric Oxide Analyzer (GE Analytical, Boulder, CO). Results were quantitated directly from the area under the curve of the chemiluminescence signal of the culture media samples and from plasma and tissue following protein removal, as previously described.^31^.

### Preparation of protein extracts and western blot analysis

Lung and PAEC protein extracts were prepared and used for Western blot analysis as previously described.^32^ Briefly, protein extracts (50 μg) were separated on 4–20% Tris-SDS gels. All gels were electrophoretically transferred to an Immuno-Blot PVDF membrane (Bio-Rad Laboratories) and blocked with 5% nonfat dry milk in Tris-buffered saline containing 0.1% Tween-20. Membranes were incubated at room temperature with primary antibody to HIF-1α (Novus Biologicals NB100-449), then washed and incubated with appropriate secondary IgG conjugated to horseradish peroxidase or fluorophore. Protein bands were then visualized with chemiluminescence (SuperSignal West Femto Substrate Kit, Pierce Laboratories, Rockford, IL) on a Kodak 440CF Image Station (Kodak, Rochester, NY) or a LI-COR Odyssey image station (Lincoln, NE). Bands were quantified using the LI-COR Image Station software. To normalize for protein loading, blots were re-probed with the housekeeping protein β-actin (Sigma Aldrich) for tissue and whole-cell lysates.

### NO assessment in cultured cells

NO levels in cultured PAECS were determined by using Diaminofluorescein-FM (DAF-FM) diacetate (ThermoFisher, Waltham, MA), a cell-permeable fluorescent dye, according to the product’s instructions. Briefly, cells were aspirated, rinsed in PBS, cultured in media with DAF-FM diacetate (final concentration 5 µM) for 30 min in the dark at 37 °C, then subjected to fluorescence microscopy. Fluorescent images were taken using an Olympus IX51 microscope equipped with a CCD camera (Hamamatsu Photonics). We quantified the average fluorescent intensities using ImagePro Plus version 5.0 imaging software (Media Cybernetics).

### Adenoviral overexpression of HIF-1α

Control PAECs (100,000 cells/well) were plated into a 6-well plate, allowed to adhere overnight, and then transduced with the adenoviral HIF-1α overexpression construct Ad-h-HIF1A (Vector Biolabs) according to the manufacturer’s specifications at a multiplicity of infection (MOI) of 100:1 in 1 ml of growth medium for 48 h. Increased HIF-1α protein in the cells was confirmed by western blot (not shown) before use in additional experiments.

## Results

### PAECs from shunt animals exhibit broad transcriptional changes associated with the stabilization of HIF-1α

**Fig. 1:**
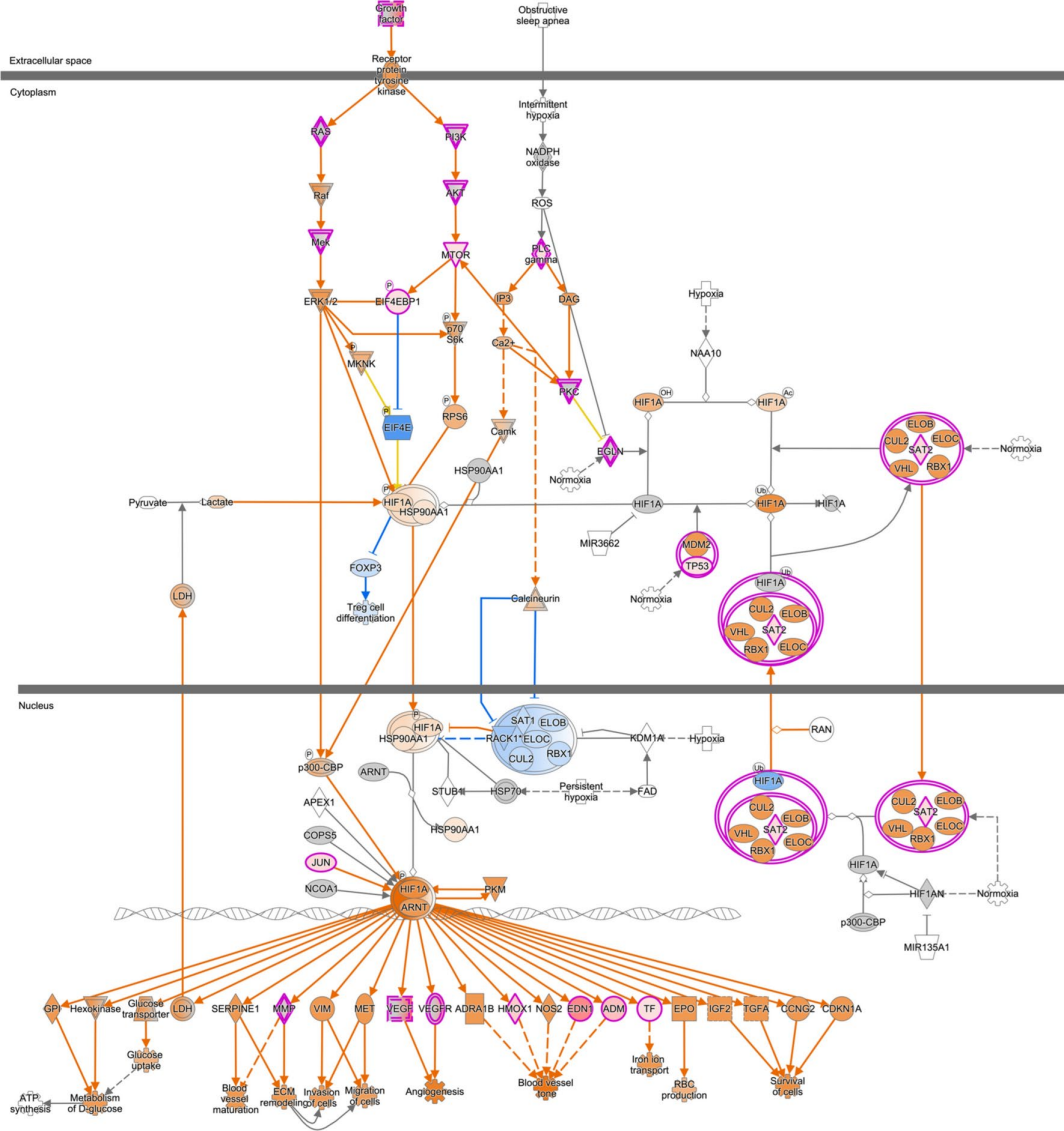
Differentially expressed transcripts mapping to the canonical HIF-1α signaling pathway in shunt PAECs, as mapped by Ingenuity Pathway Analysis Canonical pathway analysis, *p*-value of .0054 and z-score of + 3.657 indicating significant overexpression of this pathway in the shunt PAECs compared to controls. Orange = Predicted activation, Blue = Predicted inhibition, Pink = Increased measurement. N = 4 control and N = 3 shunt primary PAEC lines.

In utero placement of a surgical pulmonary-aortic conduit was performed as described previously.^5^ At 4–6 weeks of age, primary PAECs were isolated from the proximal pulmonary arteries of shunt animals and age-matched controls and submitted for RNA sequencing analysis as previously reported.^27^ The data was uploaded and analyzed using QIAGEN Ingenuity Pathway Analysis. 15,808 distinct transcripts were mapped to the IPA knowledge base, of which 1799 transcripts were identified for core and canonical pathway analyses based on differential expression with *p*-value < 0.05 corrected for multiple comparisons. Canonical pathway analysis demonstrated significant alterations in transcripts related to the HIF-1α signaling pathway, with a *p*-value of 0.0054 and z-score of + 3.657, indicating significant overexpression of this pathway in the shunt PAECs compared to controls (Fig. 1).

### Shunt PAECs and peripheral lung exhibit increased mitochondrial ROS and “normoxic” HIF-1α stabilization

**Fig. 2:**
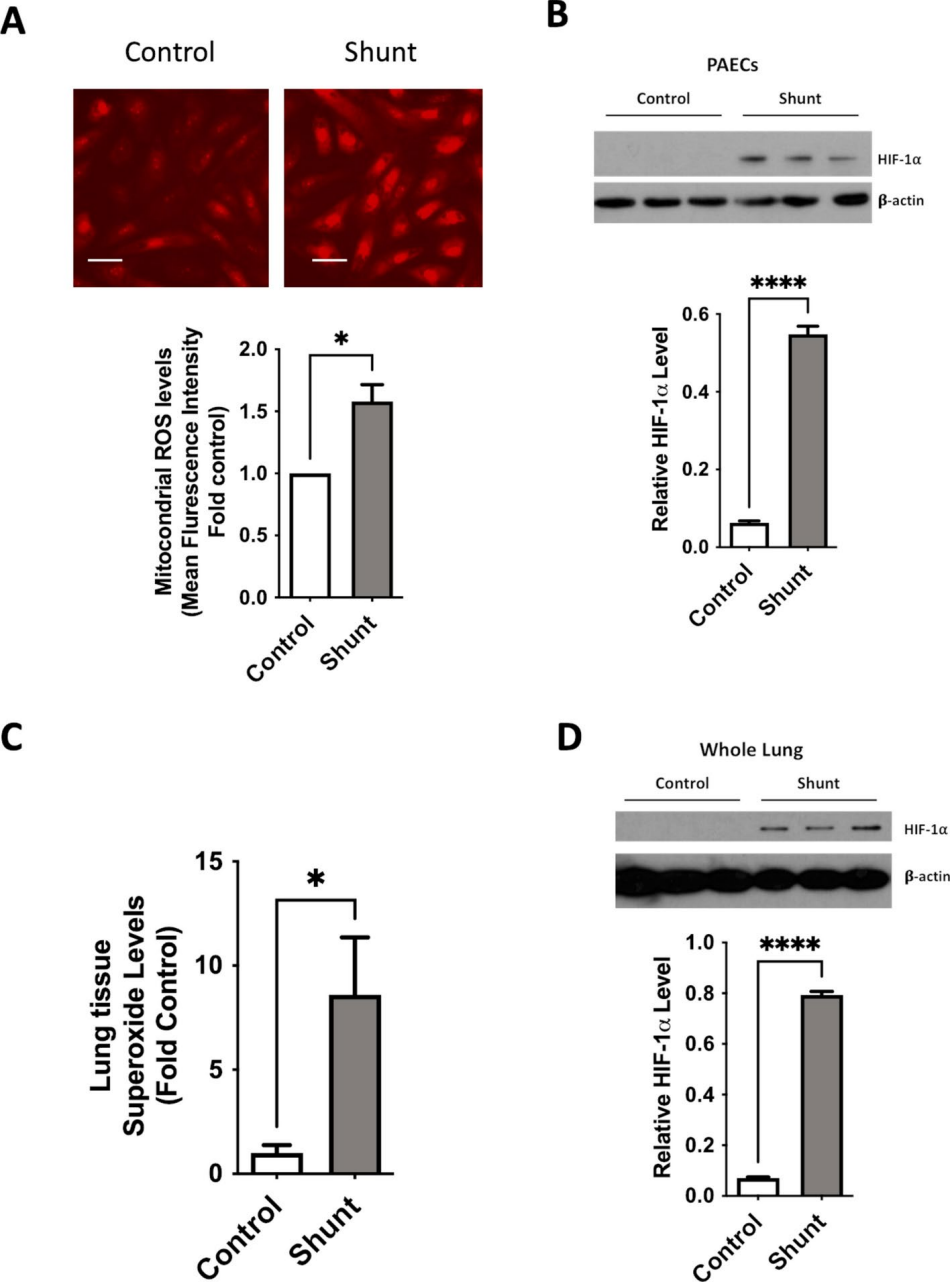
Primary PAECs and snap frozen peripheral lung tissues from shunt and control animals were compared. (**A**) Shunt PAECs exhibit higher mitochondrial ROS production based on fluorescence intensity with MitoSOX staining. Results were analyzed as control and shunt pairs, with each shunt normalized to paired control PAEC fluorescence level. N = 3 shunt and 3 control cell lines. Scale bars = 50 μM (**B**) Shunt PAECs have significantly higher levels of HIF-1α protein by western blot. N = 4 shunt and N = 5 control cell lines. (**C**) Peripheral lung tissue from shunt animals shows significantly higher levels of superoxide content by EPR analysis (**D**) Peripheral lung tissue from shunt animals also demonstrates significantly higher levels of HIF-1α protein. N = 5 shunt and N = 5 control animals for tissue studies. Western blots HIF-1α levels are normalized to β-actin as an internal loading control and expressed as band intensity ratios. All *p*-values calculated by unpaired, 2-tailed t-test with *p* < 0.05 considered significant. For bar graphs * denotes *p*-value ≤ 0.05, ** denotes *p*-value ≤ 0.01, *** denotes *p*-value ≤ 0.001 and **** denotes *p*-value ≤ 0.0001.

HIF-1α is part of a family of transcription factors canonically regulated by environmental oxygen tension. HIF-1α is continuously transcribed and translated but, under “normoxic” conditions, is subjected to post-translational hydroxylation via a family of prolyl hydroxylases (PHDs) 1,2 and 3, which target HIF-1α for ubiquitination and rapid proteasomal degradation. The activity of these PHDs and subsequent stabilization of HIF-1α has been shown to respond to direct effects of oxygen deprivation on PHD-mediated HIF-α subunit hydroxylation,^33^ but also on signaling from the mitochondria, mainly via the modulation of ROS.^34^ To confirm the results from RNA sequencing analysis, we evaluated protein levels of HIF-1α in isolated PAECs. We found that cells from the shunt animals exhibited significantly increased levels of HIF-1α protein stabilization in vitro (Fig. 2B). Evaluating for stabilizing signaling upstream, we found that PAECs from the shunt animals exhibit abnormally high production of mitochondrial ROS (Fig. 2A). Examination of whole lung tissue obtained from these animals shows similar increases in tissue superoxide levels and HIF-1α relative to control animals (Fig. 2C, D).

### Application of mechanical stimuli to PAECs alters mitochondrial ROS production and differentially affects HIF-1α stabilization

**Fig. 3:**
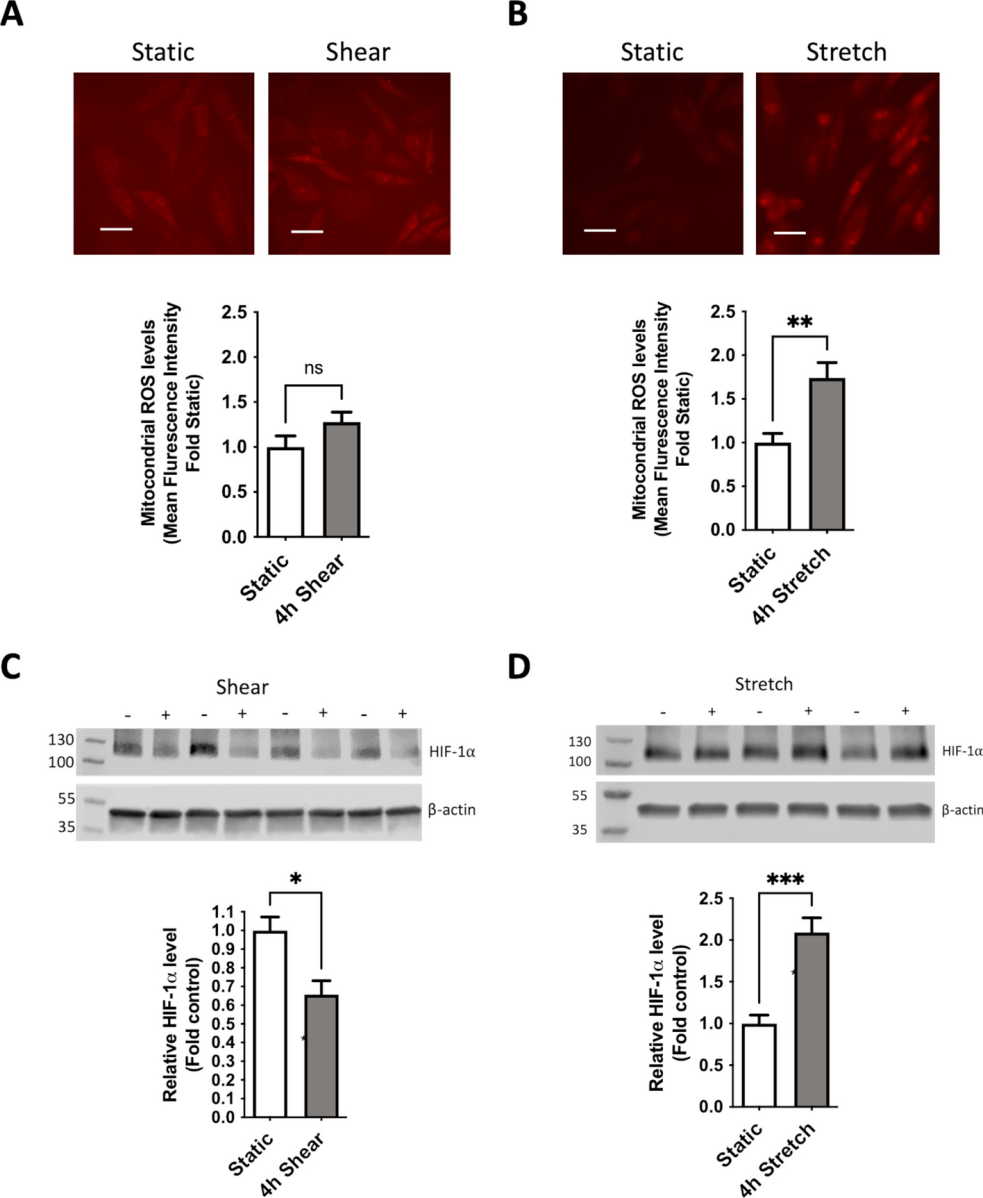
Control PAECs were variably exposed to 4 h of shear (20 dynes) or stretch (18% amplitude at 1 Hz) compared to unexposed static controls. (**A**) Shear exposure did not result in a significant increase in mitochondrial ROS by fluorescence intensity with MitoSOX staining (**B**) Stretch exposure led to a significant increase in mitochondrial ROS production compared to static PAECs. Scale bars = 50 μM (**C**) Shear exposure reduced HIF-1α protein levels, while (**D**) Stretch exposure increased HIF-1α protein levels by Western Blot relative to static controls. N = 6 exposed and unexposed groups for MitoSOX staining in shear and stretch groups, N = 4 exposed and unexposed groups for HIF-1α blots. Western blots HIF-1α levels are normalized to β-actin as an internal loading control and expressed as band intensity ratios. All *p*-values calculated by unpaired, 2-tailed t-test with *p* < 0.05 considered significant.

Notably, due to the shunting of highly oxygenated blood from the aorta, the pulmonary arterial oxygen saturations in shunt animals are increased as demonstrated by the elevated Qp/Qs ratio (Table 1), prompting us to evaluate other environmental conditions as the stabilizing forces for HIF-1α in our model. We exposed control PAECs to both shear (20 dynes) and stretch (18% amplitude at 1 Hz) stimuli and evaluated the responses in mitochondrial ROS and HIF-1α protein levels. Interestingly, the different mechanical exposures elicited notably distinct responses. Shear did not significantly increase the production of mitochondrial ROS (Fig. 3A), though it was associated with a slight but significant decrease in HIF-1α levels (Fig. 3C) compared to static conditions. However, cyclic stretch elicited a significant increase in both mitochondrial ROS (Fig. 3B) as well as HIF-1α (Fig. 3D).

**Table 1:** Baseline hemodynamic measurements and calculations from shunt animals compared to MitoQ-treated shunt animals.

Parameter	Shunt	MitoQ shunt	*P* value
HR (bpm)	146.5 ± 23.5	142.5 ± 26.4	0.74
mPAP (mm Hg)	26.6 ± 7.0	21.3 ± 7.0	0.15
Syst SAP (mm Hg)	109.9 ± 14.8	109.7 ± 20.8	0.99
Diast SAP (mm Hg)	30.8 ± 7.2	45.3 ± 16.7	0.03
mSAP (mm Hg)	58.0 ± 9.6	65.9 ± 10.7	0.14
Q LPA (ml/min/kg)	2.4 ± 0.8	2.2 ± 0.8	0.61
RAP (mm Hg)	2.4 ± 1.0	1.8 ± 1.3	0.31
LAP (mm Hg)	9.8 ± 6.4	5.6 ± 2.2	0.12
iLPAVR (mm Hg*min/ml/kg)	86.7 ± 35.0	94.3 ± 36.6	0.12
Qp:Qs	2.6 ± 0.9	2.6 ± 0.9	0.68

HR = Heart rate, mPAP = Mean pulmonary arterial pressure, Syst SAP = Systolic systemic arterial pressure, Diast SAP = Diastolic systemic arterial pressure, mSAP = Mean systemic arterial pressure, Q LPA = Flow in left pulmonary artery, RAP = Right atrial pressure, LAP = Left atrial pressure, iLPAVR = Indexed (to body weight in kg) LPA vascular resistance, Qp:Qs = Ratio of pulmonary to systemic blood flow. *P*-value by two tailed t-test for each parameter is also reported.

### Treatment of shunt PAECs with MitoQ diminishes HIF-1α stabilization and rescues NO production

**Fig. 4:**
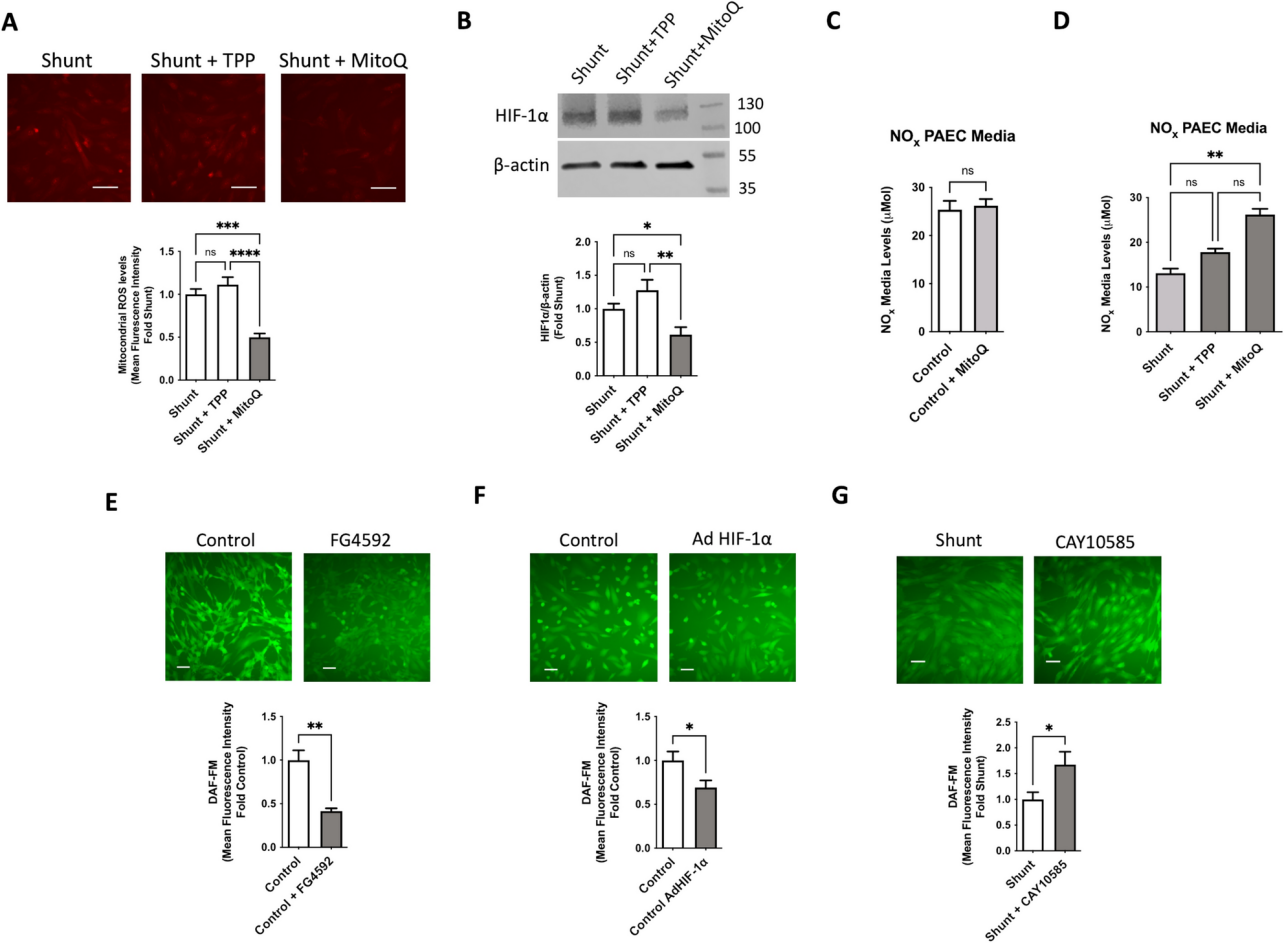
Primary PAECs derived from shunt animals treated in vitro with MitoQ (designated as Shunt + MitoQ) for 24 h exhibit: (**A**) significantly decreased mitochondrial ROS production based on fluorescence intensity with MitoSOX staining (N = 6) and (**B**) significantly decreased levels of HIF-1α protein by western blot (N = 4) compared to untreated cells. Treatment with TPP^+^ alone, the mitochondrial targeting moiety of MitoQ, does not significantly alter mitochondrial ROS or HIF-1α. (**C**) Control PAECs were cultured and treated with MitoQ and media was collected for analysis of NO_x_. MitoQ did not impact NO_x_ levels of treated compared to untreated controls. (**D**) Similarly, shunt PAECs were cultured and treated with MitoQ and TPP^+^ and media was collected for analysis. NO_x_ levels were significantly increased in media from shunt PAECs treated with MitoQ, while those treated with TPP^+^ showed no significant difference, compared to untreated shunt cells. (N = 5 for all groups). 2-way ANOVA performed with α of .05 as the significance threshold. (**E**) Control PAECs (N = 6) were treated with the prolyl-hydroxylase inhibitor FG-4592/roxadustat (100 μM for 24 h) and exhibited significantly decreased NO production by DAF FM fluorescence intensity compared to untreated control cells. (**F**) Control PAECs (N = 6) were transfected with an adenoviral vector expressing HIF-1α at a multiplicity of infection (MOI) of 100 resulting in increased cellular HIF-1α protein levels. These AdHIF1a cells also demonstrated significantly decreased NO production by DAF FM fluorescence compared to untreated control cells. (**G**) Shunt PAECs (N = 6) were treated with the HIF-1α inhibitor CAY 10,585 (10 μM for 24 h), which significantly increased NO production by DAF FM fluorescence compared to untreated shunt cells. All *p*-values calculated by unpaired, 2-tailed t-test with *p* < 0.05 considered significant.

To better understand the impact of this mitochondrial ROS-HIF-1α signaling axis and its contribution to pulmonary vascular disease, we sought to inhibit the activation of this pathway by using the antioxidant MitoQ, which specifically accumulates in the mitochondria via conjugation to the targeted lipophilic carrier triphenylphsophonium (TPP^+^). Notably, our shunt model captures an early stage of pulmonary vascular dysfunction that largely precedes the vascular remodeling of more advanced disease, but is characterized by disruption of endothelial relaxation due to derangements of endothelial nitric oxide synthase (eNOS) function and NO production. This is a consistent hallmark of both the vascular dysfunction observed in our model as well as in patients with congenital heart disease.^4,22,30^ We first demonstrated that treatment of shunt PAECs with MitoQ significantly decreased cellular levels of mitoROS (Fig. 4A) and that 24 h of therapy was sufficient to significantly decrease the levels of HIF-1α in the shunt PAECs (Fig. 4B). We then sought to evaluate the effects of treatment on NO production. We found that MitoQ therapy does not alter NO levels in control PAECs (Fig. 4C), but significantly improves the diminished NO production characteristic of shunt PAECs (Fig. 4D). While the effects of NO on stabilization of HIF-1α are established, the effects of HIF-1α on NO production are less clear. In most cell culture models, hypoxia is shown to diminish eNOS levels, though results in vivo are more variable and generally characterize hypoxia’s effects, rather than HIF activity specifically.^35^ To better characterize this observed interaction, we used pharmacologic and genetic approaches to increase HIF-1α signaling in control PAECs. Both treatment of control PAECs with the prolyl-hydroxylase inhibitor FG-4592/roxadustat and adenoviral overexpression of HIF-1α resulted in decreased cellular production of NO by DAF-FM fluorescence (Fig. 4E, F). Similarly, treatment of shunt PAECs with the HIF-1α inhibitor CAY 10,585, restored cellular NO production in a similar manner to MitoQ (Fig. 4G).

### MitoQ therapy administered to shunt lambs attenuates increases in endothelial ROS and inhibits HIF-1α stabilization

**Fig. 5:**
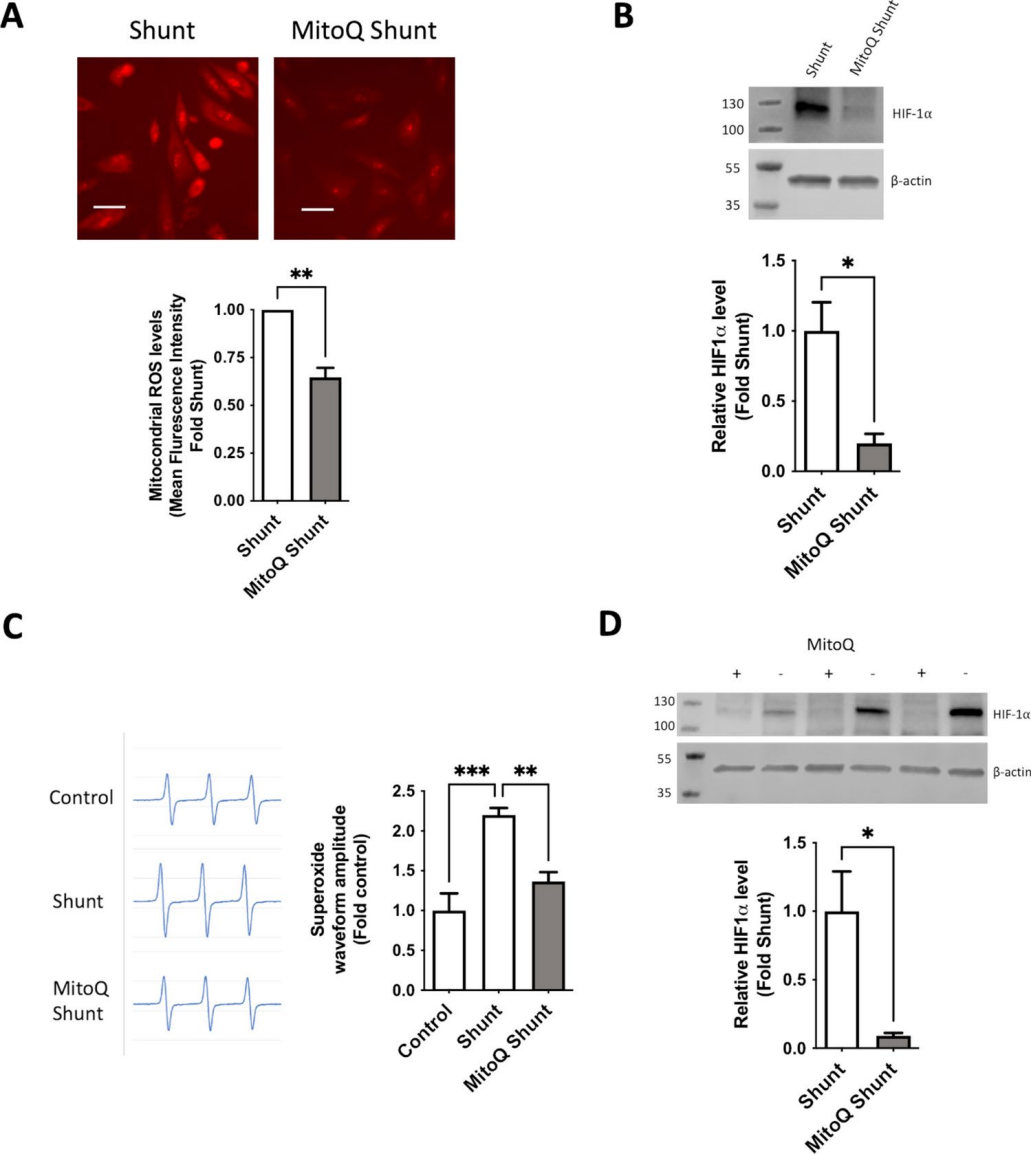
Shunt animals were treated daily with oral MitoQ. Primary PAEC cell lines were derived, and peripheral lung tissues collected and snap frozen from treated (note that all tissues and PAECs derived from these treated animals are labelled “MitoQ Shunt”) and untreated shunt animals for analysis. (**A**) MitoQ shunt PAECs exhibit lower mitochondrial ROS production than shunt PAECs based on fluorescence intensity with MitoSOX staining. Results were analyzed as control and shunt pairs, with each shunt normalized to paired control PAEC fluorescence level. N = 3 shunt and N = 3 control cell lines. (**B**) MitoQ shunt PAECs (N = 4) have significantly lower HIF-1α protein levels than shunt cells (N = 5) by Western Blot. (**C**) Peripheral lung tissue from MitoQ shunt animals (N = 4) demonstrates lower superoxide levels than shunt animals (N = 4) and no different from lung tissue from physiologically normal control animals (N = 4) by EPR with representative waves included for each group. Analysis was performed by 2-way ANOVA with α of .05 as the significance threshold. (**D**) Peripheral lung tissue from MitoQ shunt animals (N = 4) has significantly lower levels of HIF-1α protein than untreated shunts. Western blots HIF-1α levels are normalized to β-actin as an internal loading control and expressed as ratios of band intensity. All *p*-values calculated by unpaired, 2-tailed t-test with *p* < 0.05 considered significant.

After demonstrating a significant biologic effect of mitochondrial ROS scavenging on the PAECs in vitro, we evaluated the effects of systemic mitochondrial antioxidant therapy in our animal model. Shunt animals were treated daily with oral MitoQ at ~ 2 mg/kg starting immediately after birth until physiologic evaluation, tissue collection, and isolation of PAECs at 4–5 weeks of age and compared with untreated shunt animals as controls. Baseline physiologic parameters of both the MitoQ treated and untreated shunt animals are presented in Table 1. Like our findings of acute in vitro treatment, cultured primary PAECs derived from animals who received daily mitoQ therapy (MitoQ Shunt) showed significant decreases in baseline mitochondrial ROS production (Fig. 5A) and HIF-1α stabilization (Fig. 5B). Similar effects were observed in whole lung tissue, which demonstrated decreased superoxide and HIF-1α levels in the MitoQ treated shunt animals compared to untreated controls (Fig. 5C, D).

### MitoQ therapy administered to shunt lambs restores endothelial NO production

**Fig. 6:**
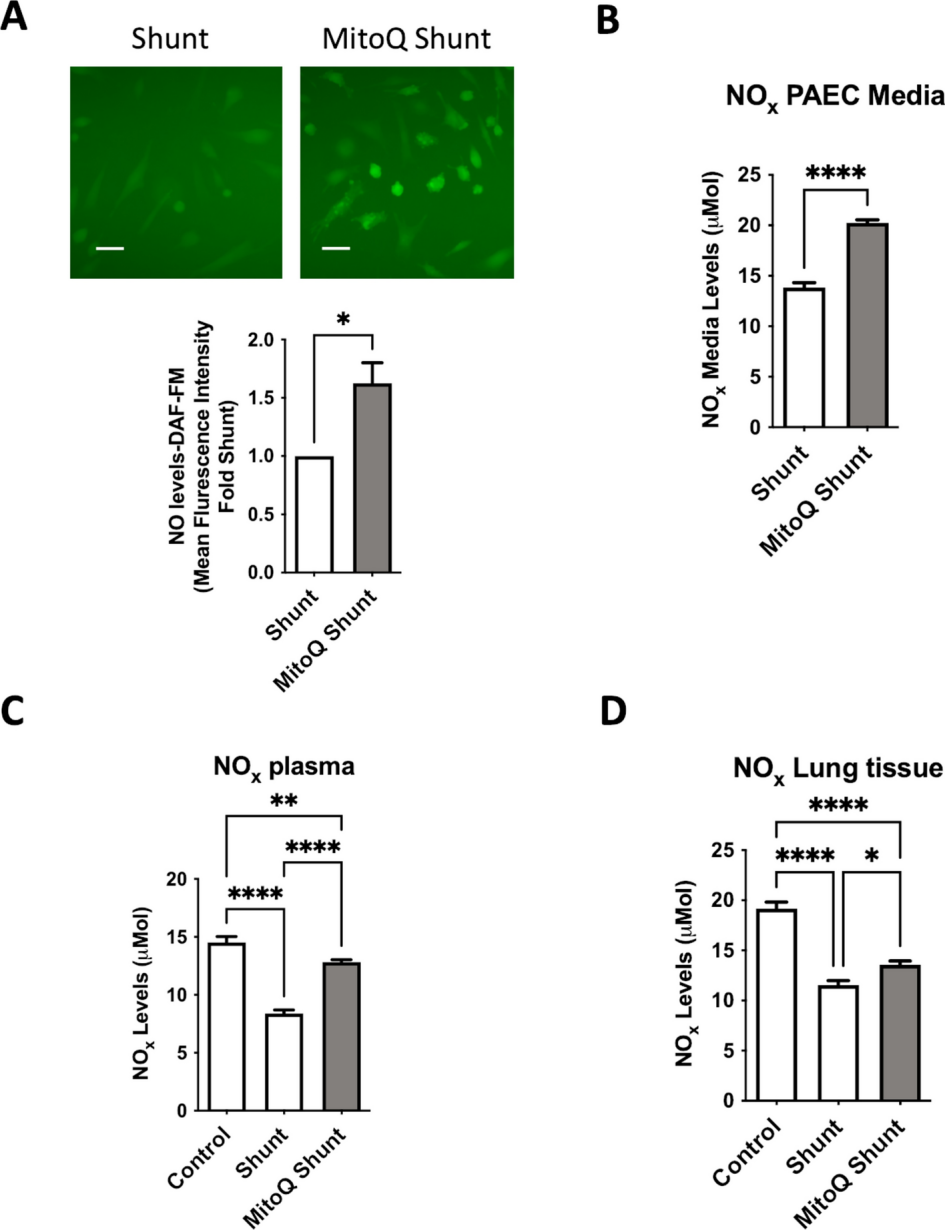
Primary PAEC cell lines, serum and peripheral lung tissue derived from treated MitoQ shunt and untreated shunt animals were evaluated for NO levels and production. (**A**) MitoQ shunt PAECs exhibit increased NO production compared to untreated shunt cells by DAF FM fluorescence intensity. Results analyzed as control and shunt pairs, with each shunt normalized to paired control PAEC fluorescence level. N = 3 shunt and N = 3 control cell lines. (**B**) Culture media from MitoQ shunt PAECs contains higher levels of NO_x_ than media from untreated shunt cells. (N = 3 MitoQ shunt and N = shunt cell lines) (**C**) Plasma samples and (**D**) peripheral lung tissue from MitoQ shunt animals have significantly higher NO_x_ levels than corresponding samples from untreated shunt animals, though not as high as physiologically normal control animals. (N = 3 MitoQ shunt, shunt, and normal control animals per group performed on samples in triplicate). *p*-values for A&B calculated by unpaired, 2-tailed t-test with *p* < 0.05 considered significant. Analysis for C&D was performed by 2-way ANOVA with α of .05 as the significance threshold.

To assess the physiologic effects of MitoQ therapy in the shunt animals, we again sought to examine pulmonary endothelial NO production. Evaluating primary PAECs from the MitoQ-treated shunt animals compared to untreated; we demonstrated increased levels of basal NO production by DAF fluorescence staining of the cells (Fig. 6A), as well as by measurement of bioavailable NO (NO_x_) released into the cell culture media (Fig. 6B). Correspondingly, evaluation of NO_x_ in the serum and lung tissue of MitoQ shunt, untreated shunt, and physiologically normal control animals, we found that MitoQ therapy significantly increased NO_x_ levels compared to untreated shunt animals, though did not fully restore it to control levels (Fig. 6C, D).

### MitoQ therapy administered to shunt lambs improves endothelial function in vivo

**Fig. 7:**
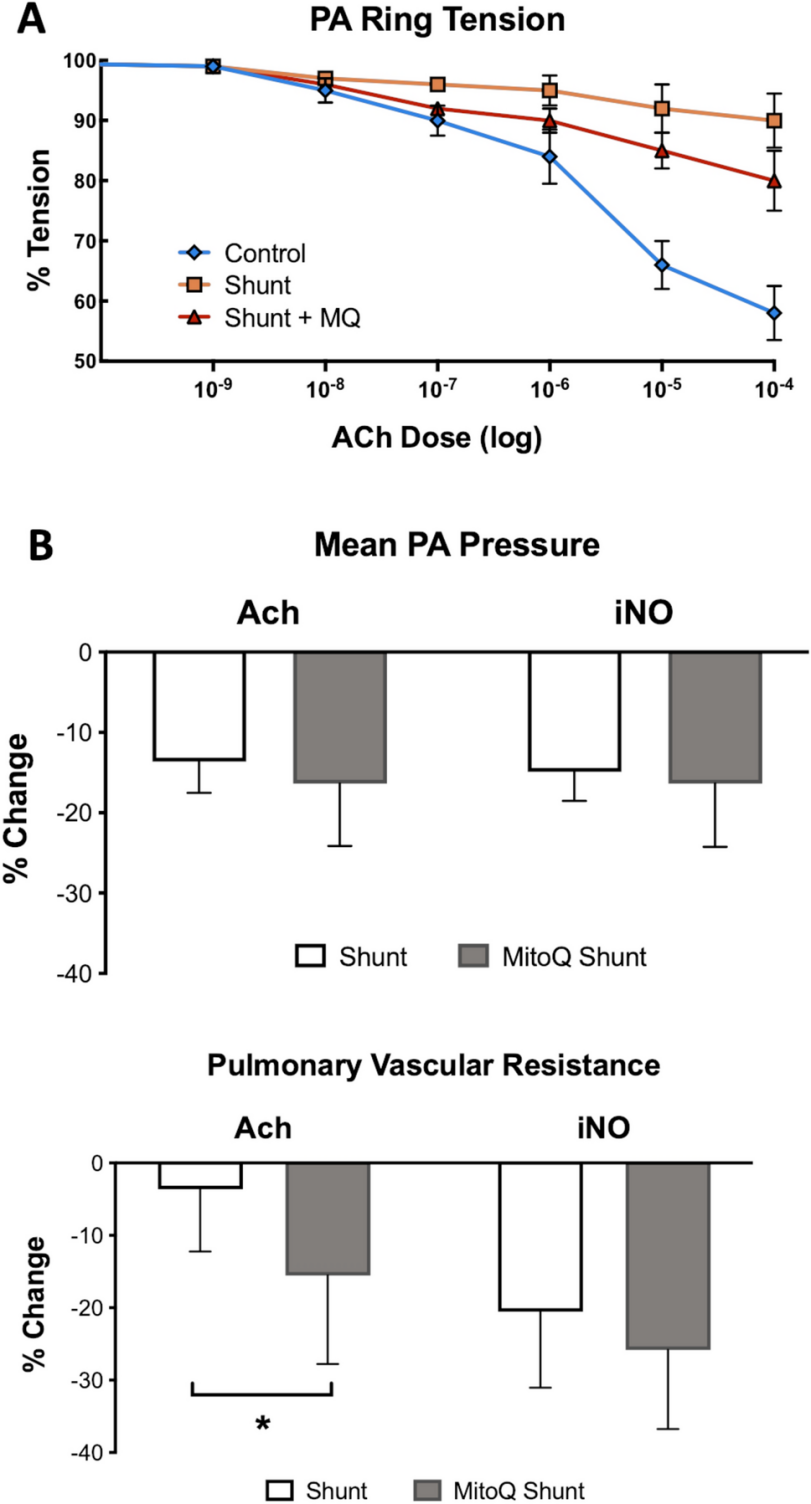
Isolated PA rings from 4-5th generation vessels were mounted on a pressure myograph and pre-constricted with norepinephrine, then assessed for response to the endothelial and NO-dependent vasodilator acetylcholine (Ach). (**A**) Rings from the MitoQ shunt animals exhibited significantly improved relaxation compared to untreated shunts in response to Ach, though not at the level of normal control animals. N = 7 animals in each group. Analysis was performed by 2-way ANOVA for each dose of Ach with α of .05 as the significance threshold. * denotes significant difference between shunt and control, # between MitoQ shunt and control, and & between MitoQ shunt and shunt. Changes in mean PA pressure and pulmonary vascular resistance (PVR) were calculated in response to intravenous Ach and inhaled NO (iNO) were assessed in anesthetized and ventilated MitoQ shunt and shunt animals. (**B**) MitoQ shunt animals exhibit a significantly larger decrease in PVR in response to the endothelium-dependent vasodilator Ach compared to untreated shunt animals. The endothelium-independent vasodilator iNO does not demonstrate a significant decrease in PVR between groups. Data is presented as percent deviation from the immediate pre-treatment baseline. N = 10 MitoQ shunts and untreated shunts for physiologic analysis.

To verify the physiologic relevance of this rescue in NO production, we isolated pulmonary artery segments for vasoreactivity evaluation. 4-5^th^ generation PA rings were mounted on a pressure myograph and pre-constricted with norepinephrine, then assessed for response to the endothelial and NO-dependent vasodilator acetylcholine (Ach). As seen in Fig. 7A, rings from the MitoQ-treated shunt animals exhibited significantly improved relaxation compared to untreated shunts in response to Ach. However, the vasodilatory response of MitoQ shunts did not improve to the level of normal controls. Finally, we assessed pulmonary vascular responses in vivo in anesthetized ventilated animals. The changes in mean PA pressure and pulmonary vascular resistance (PVR) were calculated in response to intrapulmonary Ach and inhaled NO (iNO), which acts at the level of the vascular smooth muscle directly. We have previously shown that shunt animals have impaired endothelial-mediated vasodilation to Ach but intact response to iNO. MitoQ significantly improves the pulmonary vascular relaxation to Ach in treated compared to untreated shunts, as evidenced by the enhanced reduction in PVR in these animals (Fig. 7B). The lack of significant difference in response to the endothelium-independent vasodilator iNO confirms that the differential response observed is related to endothelial function.

## Discussion

Mitochondria serve as central coordinators of cellular metabolism, integrating and balancing environmental inputs such as nutrient availability and oxygen tension against cellular requirements for homeostasis and growth. Subversion and disorder of these processes has been shown to widely facilitate the vascular dysfunction and proliferative derangements that characterize PAH. This commonly includes increased glycolysis and suppression of aerobic metabolism and the mitochondrial electron transport chain, alterations in mitochondrial membrane potential and ROS production, and stabilization of the hypoxia-inducible factors HIF-1α and HIF-2α despite “normoxic” cellular oxygen environments.^19,36^ Consistent with these observations, previous work with our shunt model of pulmonary vascular disease has demonstrated a recurrent pattern of vascular mitochondrial dysfunction. The pulmonary lymphatic endothelium, PA endothelium, and PA smooth muscle all exhibit suppression of basal aerobic metabolism, with alterations in mitochondrial membrane potential and mitochondrial ROS relative to normal controls.^6–8^ The PA endothelium exhibits fragmentation of mitochondrial networks, and both the lymphatic and PA endothelial cells exhibit stabilization of HIF-1α.^7,8^

The HIFs are heterodimeric transcription factors primarily regulated post-transcriptionally via targeted degradation of the α subunits. Constitutive PHD-mediated hydroxylation of proline residues within the α subunits allows binding of the von-Hippel Lindau (VHL) protein and subsequent ubiquitination and proteasomal destruction. The hydroxylation reaction utilizes molecular O_2_ as a cofactor, and direct substrate limitation in cellular hypoxia can confer the hypoxic responsiveness that is the namesake of these factors.^34,37^ However, elegant work by multiple groups has demonstrated that the hypoxic stabilization of HIF α subunits is also regulated directly by mitochondrial function and is mediated by modulation of mitochondrial ROS production.^19^ In this study, we demonstrate that PA endothelial cells from our shunt animals exhibit increased mitochondrial ROS production relative to physiologically normal control animals and that scavenging these ROS with the mitochondrially-targeted antioxidant MitoQ suppresses the endothelial stabilization of HIF-1α. Notably, our animal model is based on the shunting of highly oxygenated blood from the aorta into the relatively de-oxygenated blood of the pulmonary arterial system. This results in an increase in PA oxygen content that would be expected to suppress hypoxic signaling cascades. Therefore, given that our model is grounded in the hemodynamic precipitants of vascular disease, we looked to aberrant mechanical forces, rather than environmental oxygen tension, to explain the alterations in mitochondrial ROS production and HIF-1α stabilization we observe.

We show here that even short exposures of PAECs to specific mechanical stimuli can induce significant changes in mitochondrial ROS generation and HIF-1α signaling. This aligns with an increasing body of experimental evidence demonstrating structural and functional responses of mitochondria to external mechanical stimuli. Several groups have shown that cells respond to alterations in the stiffness of their surrounding extracellular matrix (ECM) by increasing dynamin-related protein 1 (Drp1) mediated mitochondrial fission, with associated alterations in mitoROS.^11,38,39^ Electron microscopy of pressurized optical neural cells in the setting of glaucoma has shown mitochondrial morphodynamic changes, including fission, swelling, and cristae depletion. In contrast, the application of high hydrostatic pressure to HUVECs in vitro has been shown to induce elevations in mitochondrial membrane potential and mitochondrial ROS production, with suppression of Drp1 and induction of the mitochondrial fusion-related protein OPA1.^16^ Various mitochondrial responses have been linked to shear stress, as outlined in a recent review describing shear-induced alterations in fission and fusion, MMP, mitochondrial ROS, and aerobic metabolism.^14^ HIF-1α stabilization in response to disturbed flow patterns has similarly been described along with metabolic alterations, though not directly linked to mitochondrial function or morphodynamics changes.^40^ Notably, the specifics of the described mitochondrial responses are disparate and depend on the cell type, nature, and magnitude of shear exposure, with the differential responses to laminar versus disturbed flow serving as a paradigm of this context dependence.^14^ Perhaps one of the best examples of this phenomenon is observed in the setting of mechanical strain rather than shear, as described in elegant studies by Bartolak-Suki and Suki. Exposing vascular smooth muscle cells (VSMCs) to cyclical area strain across a range of magnitudes spanning from static to supraphysiologic, they demonstrated that the VSMCs maintained optimal aerobic metabolic capacity and minimal mitochondrial ROS production within the physiologic range. In contrast, deviation either above or below this range led to fragmentation of mitochondrial networks, diminished aerobic capacity, and elevated mitochondrial ROS.^17^.

**Fig. 8:**
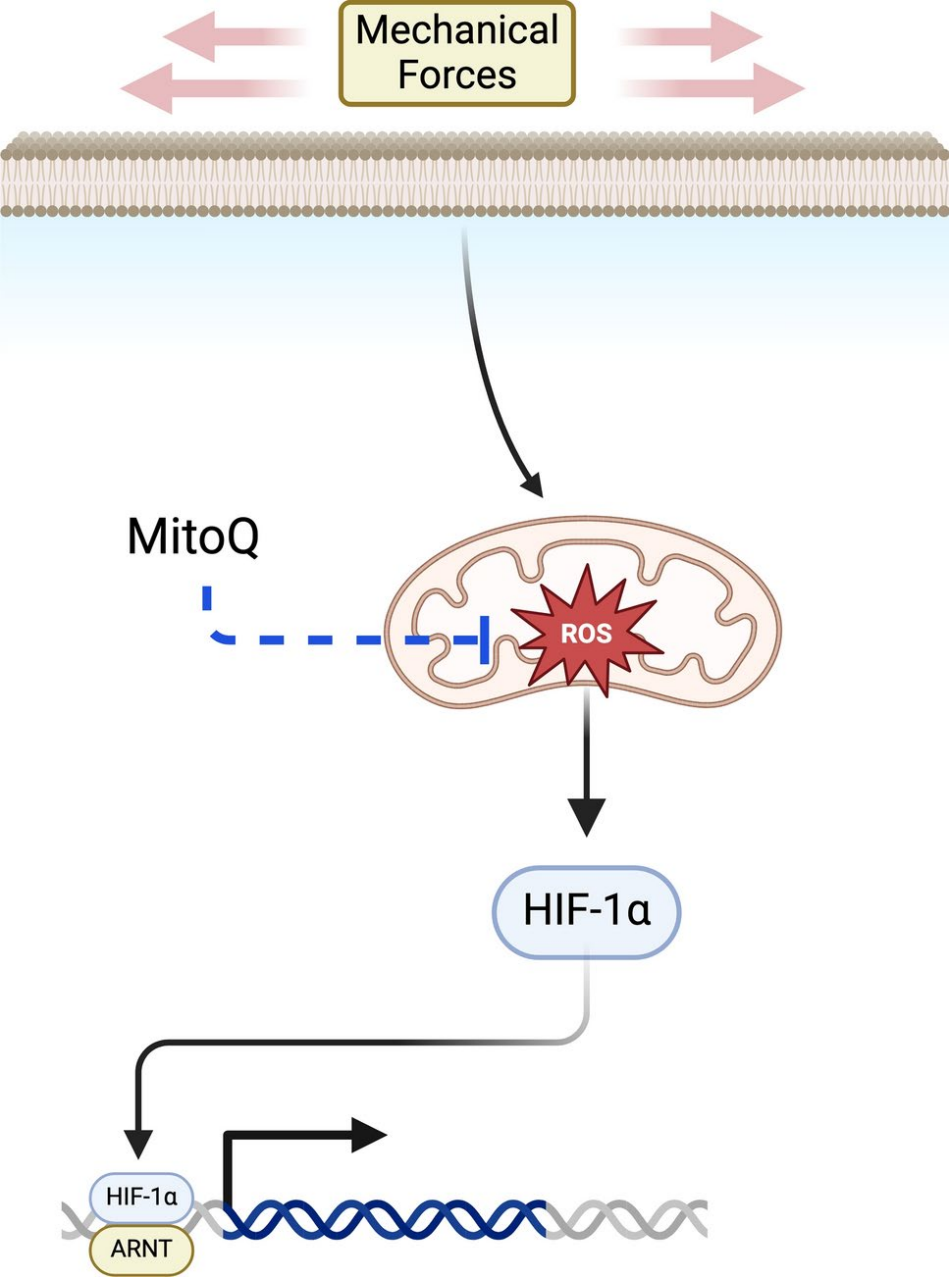
Illustrated graphic depicting the central pathway proposed in this manuscript: abnormal hemodynamic forces acting on the vascular endothelium induce mitochondrial responses, including an increase in ROS production, that stabilizes the redox sensitive transcription factor HIF-1α. Created in BioRender. Boehme, J. (2025) https://BioRender.com/t77h924.

These accumulated findings strongly support the idea that cellular mitochondria are highly attuned to the mechanical forces that constitute their specific physiologic environment and react rapidly to any deviations from their norm, often through mechanisms that influence the production of mitochondrial ROS. However, the downstream consequences of such mitochondrial responses remain widely unknown. Given our findings here and previous work with the shunt model, we believe that the pervasive mitochondrial dysregulation observed in the vasculature of these animals represents an integrated and direct mitochondrial response to abnormal hemodynamic forces, with the stabilization of HIF-1α representing a conserved mitochondrial engagement of downstream redox-sensitive signaling pathways. We show in this manuscript that abnormal hemodynamic forces acting on the vascular endothelium induce mitochondrial responses, including an increase in ROS production that stabilizes the redox sensitive transcription factor HIF-1α (Fig. 8). We further demonstrate that the stabilization of HIF-1α in the PA endothelium inhibits endogenous NO production, and that treatment with a mitochondrial antioxidant, both in vitro and in vivo, attenuates HIF-1α stabilization and improves endothelial NO production. Our shunt model notably captures a stage of pulmonary vascular disease that precedes the significant proliferative vasculopathy of advanced disease states and corresponds to an early and seemingly reversible stage of disease in patients which is characterized physiologically by the dysfunctional endothelial production of NO. Our group and others have previously shown that this phenotype is complex, and interrelated with endothelial abnormalities of metabolic function, organelle trafficking and localization, cellular signaling cascades, transcriptional changes, and various post-translational modifications of eNOS.^4,22,23,27^ Given this, it is perhaps unsurprising that MitoQ does not completely restore endothelial function to control levels. However, we believe that our findings of mitochondrial and metabolic dysfunction, altered redox signaling, and “normoxic” HIF stabilization represent a consistent through line linking the pathophysiology of early and advanced disease states. Importantly, we show here that the use of a mitochondrial antioxidant can interrupt this signaling axis at an early stage of disease and restore meaningful vascular physiologic function in vivo through restoration of endothelial NO production.

Furthermore, a model placing mitochondria and mitochondrial ROS as central nodes in pathologic mechanotransductive signaling relays suggests significant potential for biologic overlap and interactions with hypoxic states. Hypoxia itself is a ubiquitous risk factor for the development of pulmonary vascular disease and serves as a critical pathologic stimulus in numerous animal models of PH. In terms of its clinical significance, a recent study from the North American PPHNet identified Group 3, pertaining to hypoxia and lung disorders, to be the most common cause of PH amongst registered children.^41^ There is limited evidence that begins to explore the existence of such interactions. Work by Giedt et al. utilized variable shear and hypoxic exposures investigating ischemic reperfusion to demonstrate that hypoxic pre-exposure primed endothelial cells to respond to subsequent shear with mitochondrial fission and a significant increase in mitochondrial ROS, surpassing the response to either stimulus in isolation.^42^ In the clinical literature, several recent publications have noted that in premature infants with bronchopulmonary dysplasia, both the presence and duration of high-pressure left-to-right shunting via a patent ductus arteriosus were positively associated with the development of pulmonary hypertension.^43,44^ The potential for shared, possibly synergistic, pathophysiologic mechanisms raises fascinating prospects concerning common therapy for both hypoxic and hemodynamic etiologies of PH via modulation of mitochondrial redox signaling. However, it must be recognized that mitochondrial ROS play essential roles in normal physiologic and developmental processes, and further investigation is required to safely leverage these therapies, particularly in a pediatric context of rapid growth and development.

## Supplementary Information

Supplementary Information.

## Data Availability

The datasets analyzed in the current study are available from the corresponding author on reasonable request. The RNA sequencing datasets referenced in Fig. 1 have been made previously publicly available in conjunction with the publication of reference 27 in this manuscript (Kameny, R. J. et al*.* Ovine models of congenital heart disease and the consequences of hemodynamic alterations for pulmonary artery remodeling. *Am. J. Respir. Cell Mol. Biol.* **60**, 503–514 (2019).)

## Declarations

### Generative AI

Generative AI technology was not used in the composition of the manuscript or any supporting content.
