# Supplementary material for: Mechanotransductive stabilization of HIF-1α is inhibited by mitochondrial antioxidant therapy in the setting of pulmonary overcirculation
Source: Sci Rep. 2025 May 10;15:16320. doi: 10.1038/s41598-025-99062-0 (PMC12065877; doi:10.1038/s41598-025-99062-0)
Supplement: Supplementary file 1 — Supplementary Information. [file 41598_2025_99062_MOESM1_ESM.pdf]

Figure 2B&D

PAEC HIF-1α control vs shunt

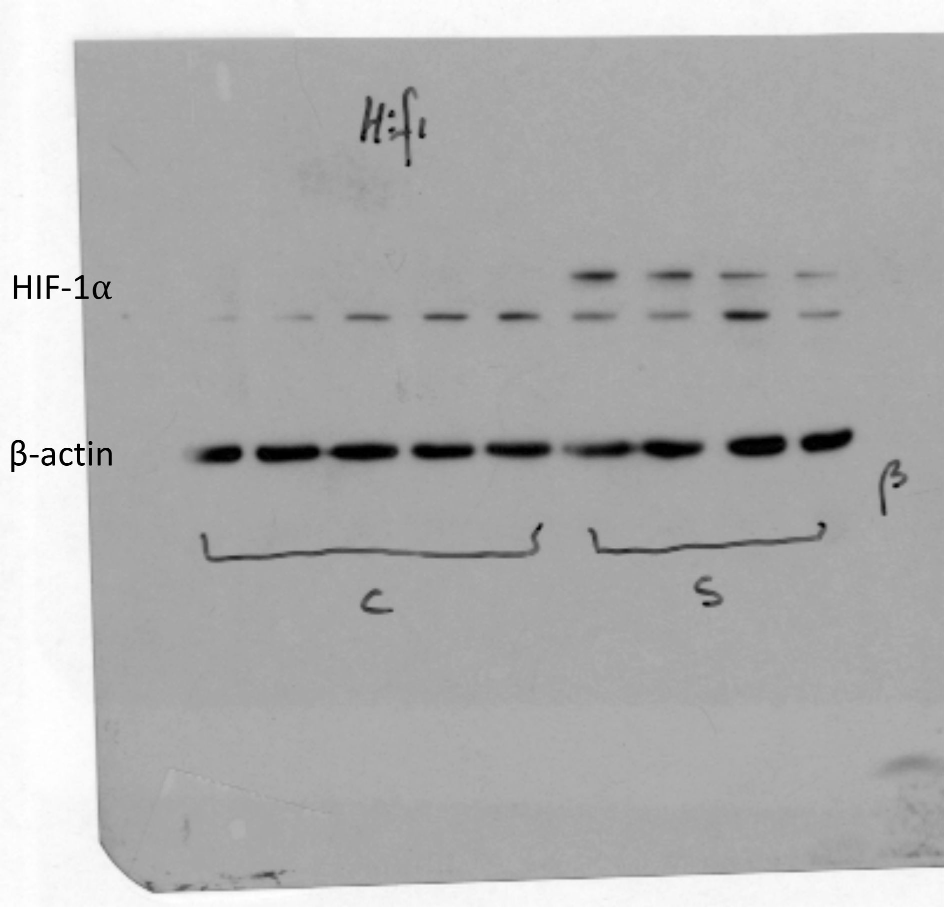

Whole lung tissue HIF-1α control vs shunt

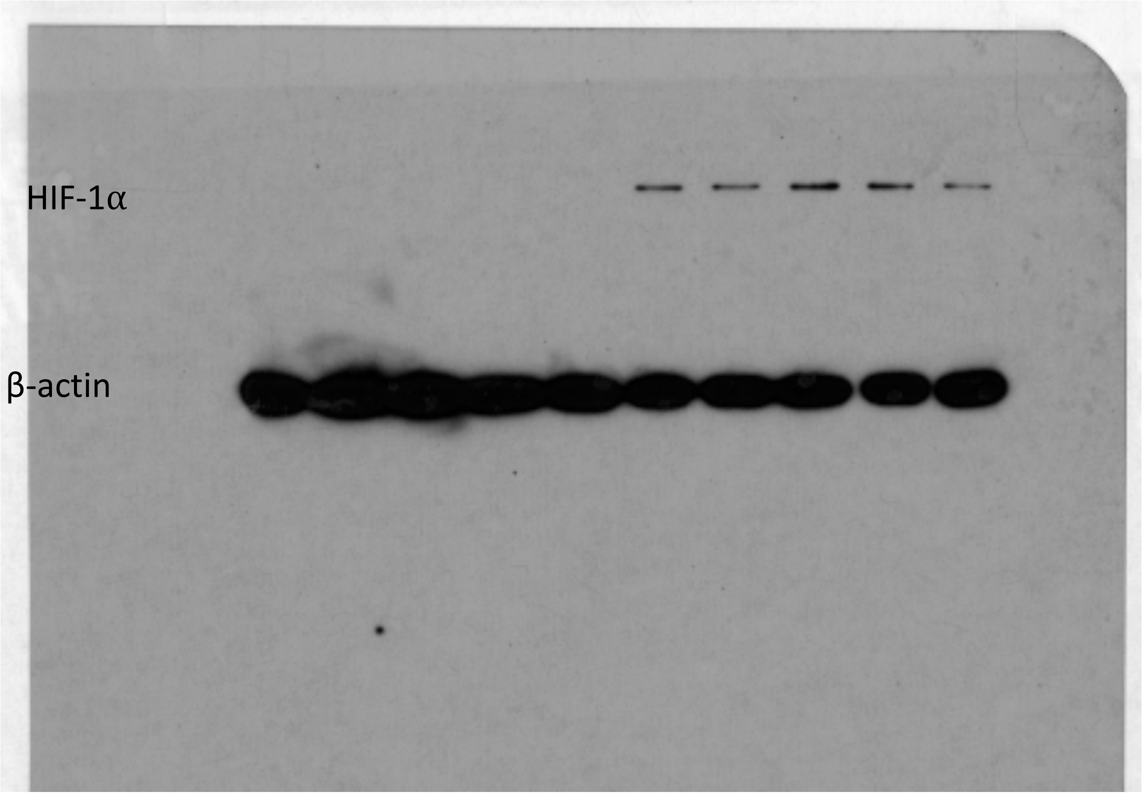

Figure 3C

PAEC HIF-1α shear and stretch exposure

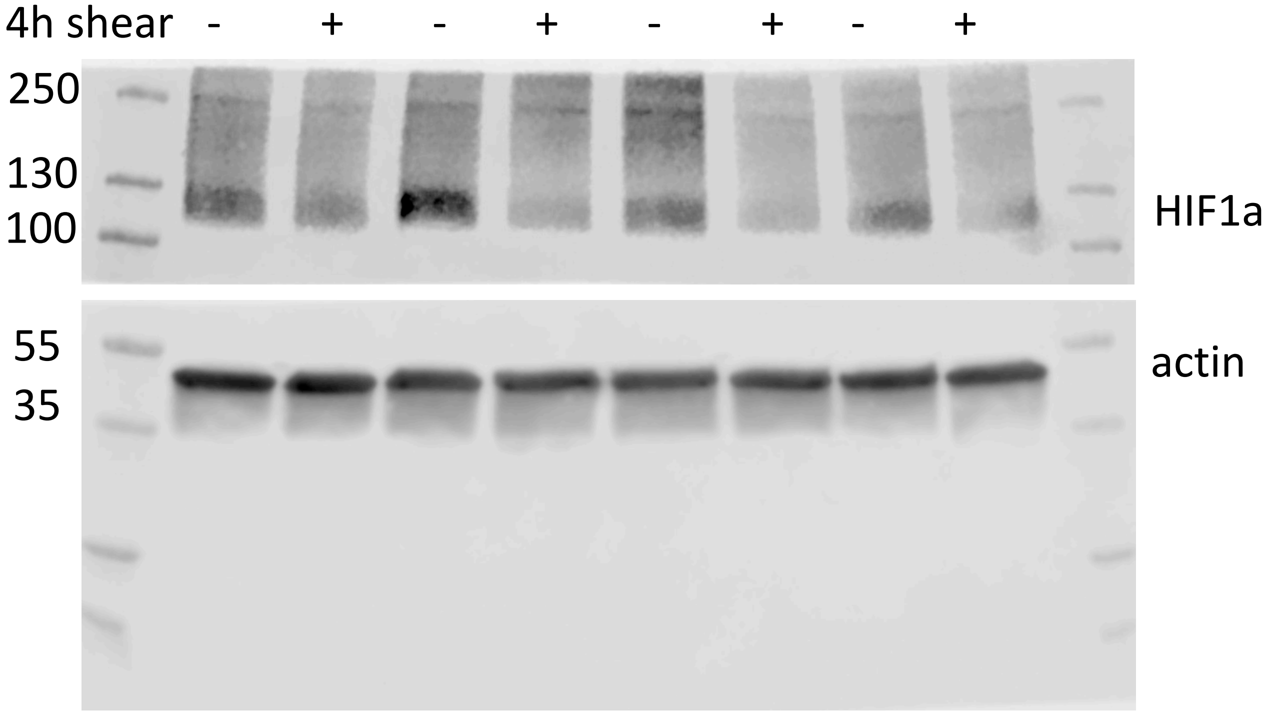

Figure 3D

PAEC HIF-1α shear and stretch exposure

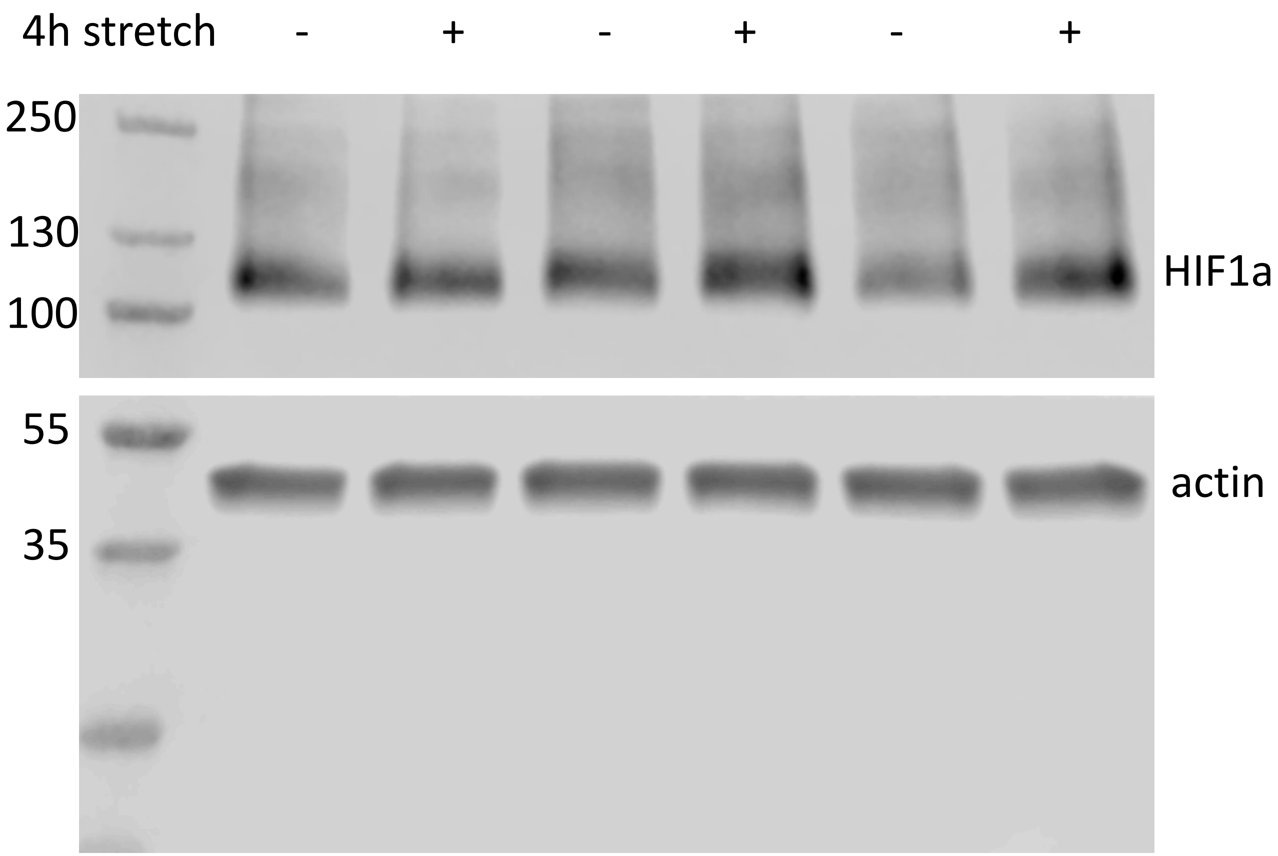

Figure 4B
PAEC HIF-1α expression TPP and MitoQ treatment

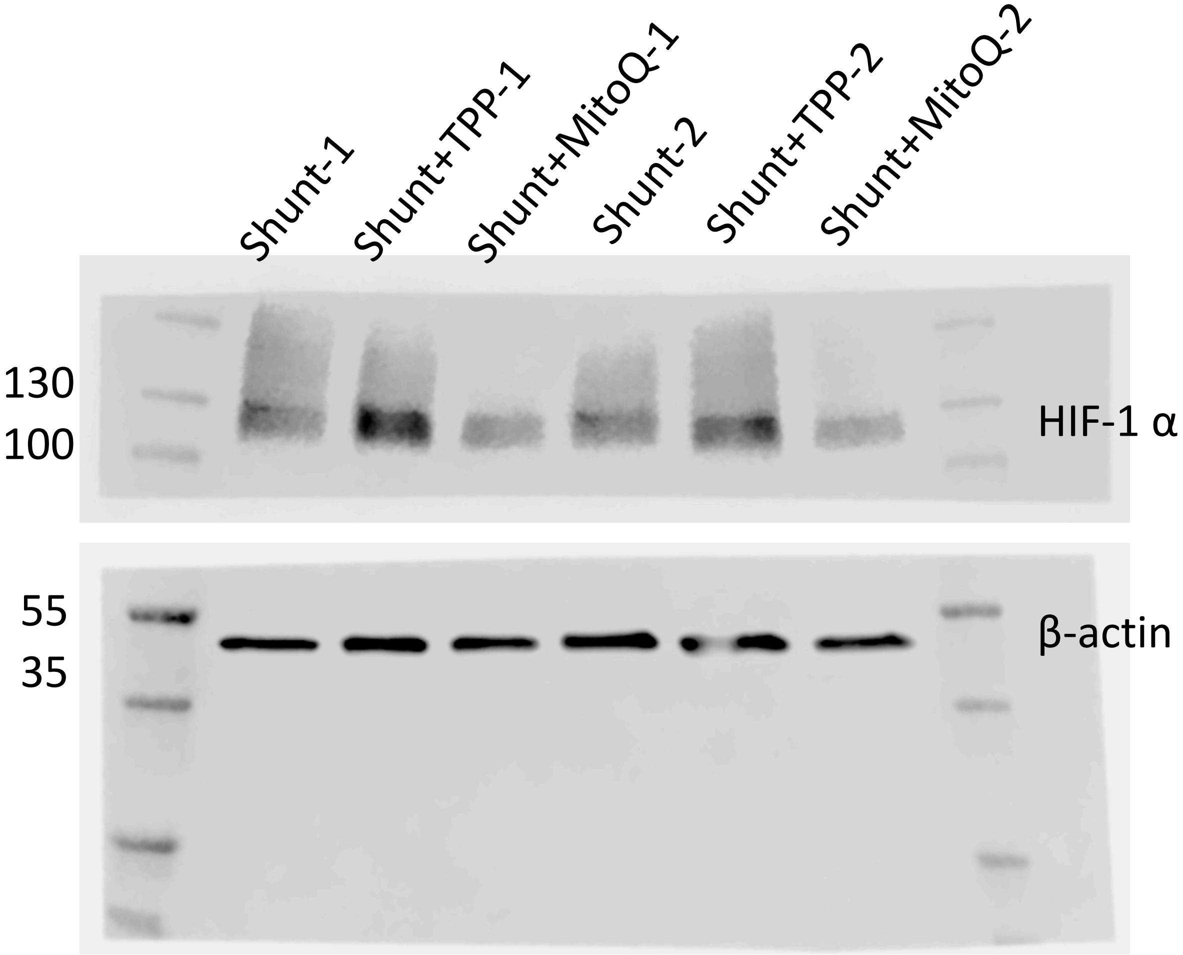

Figure 5B
PAEC Representative Image

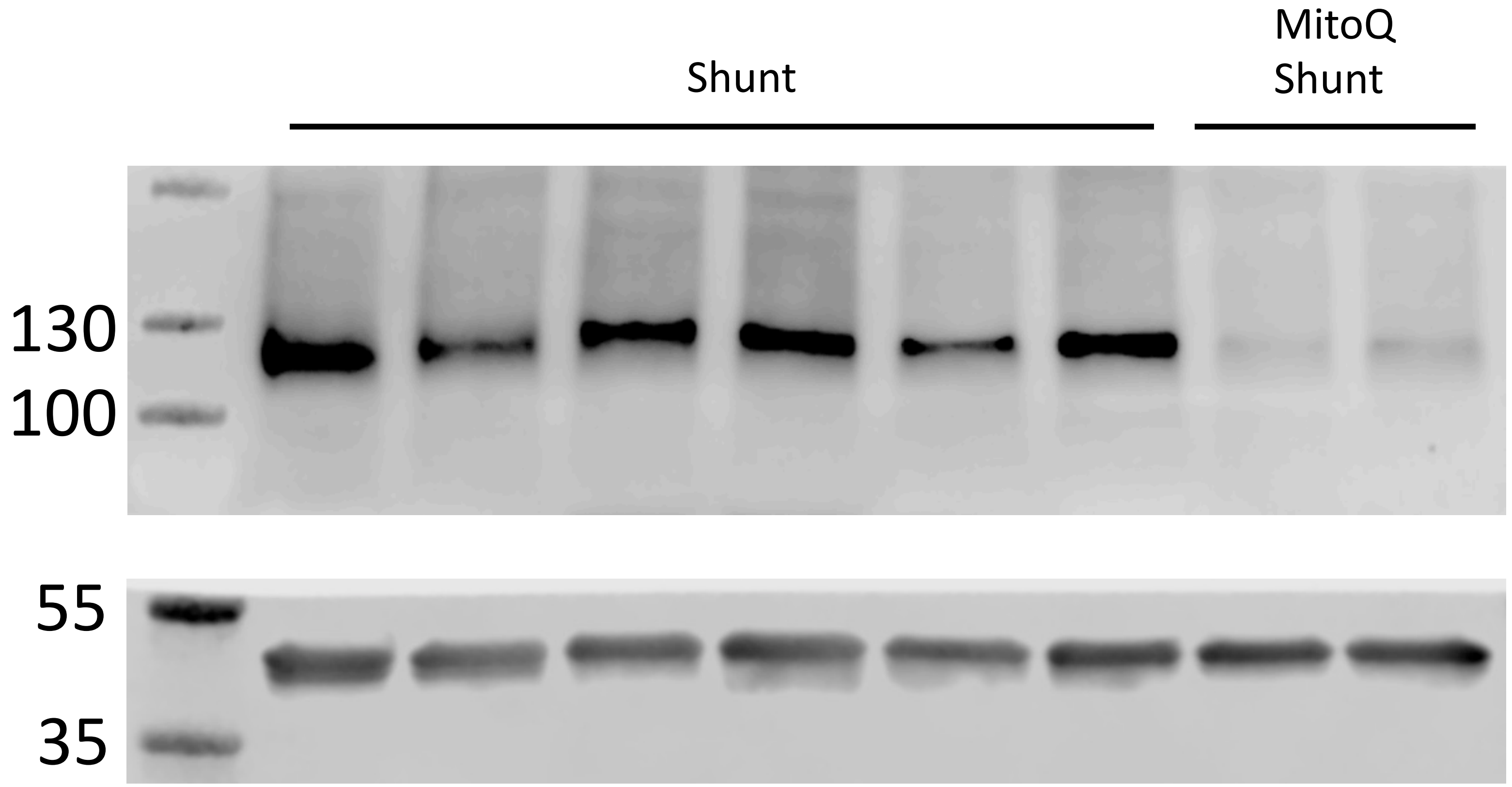

Figure 5D

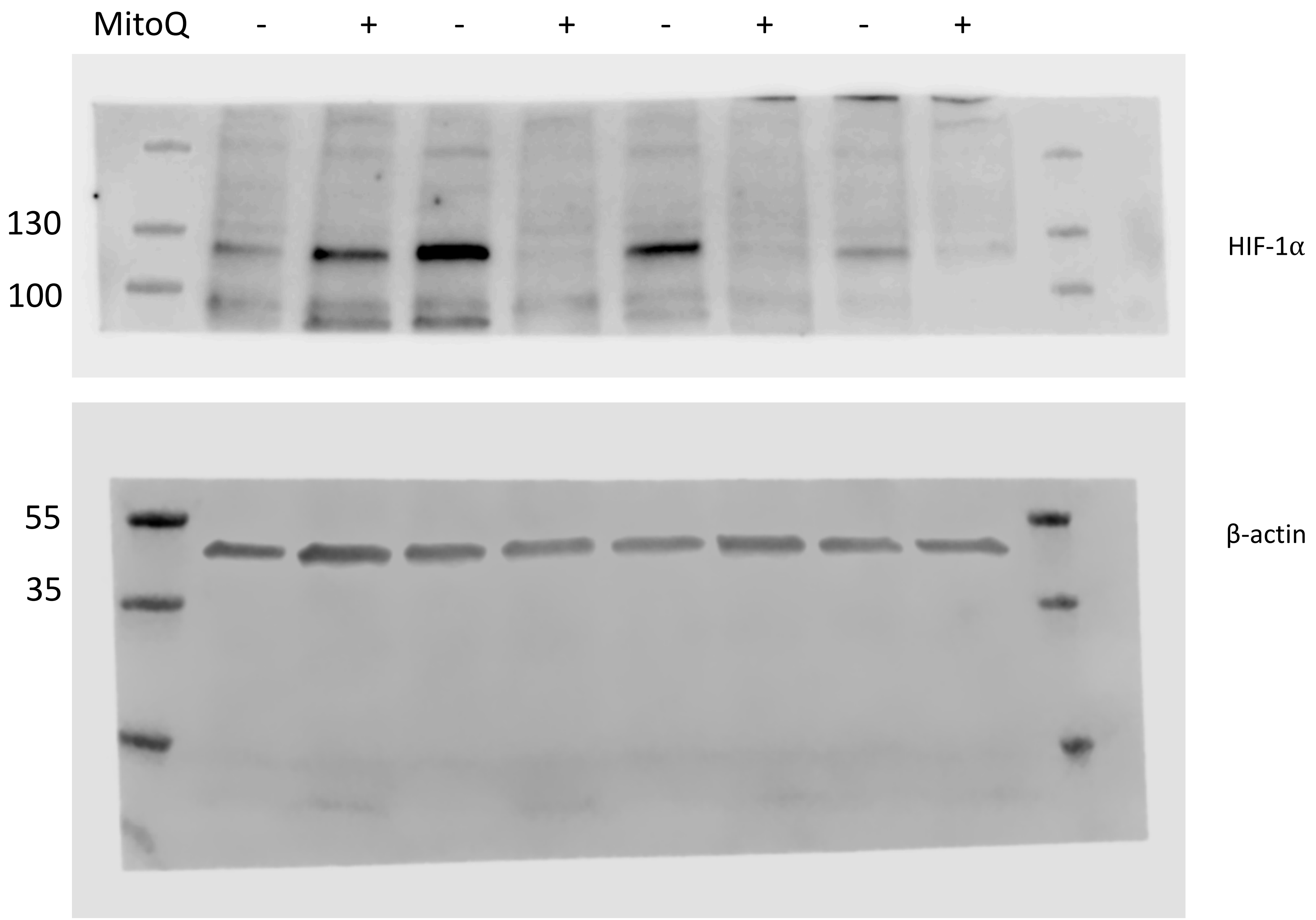

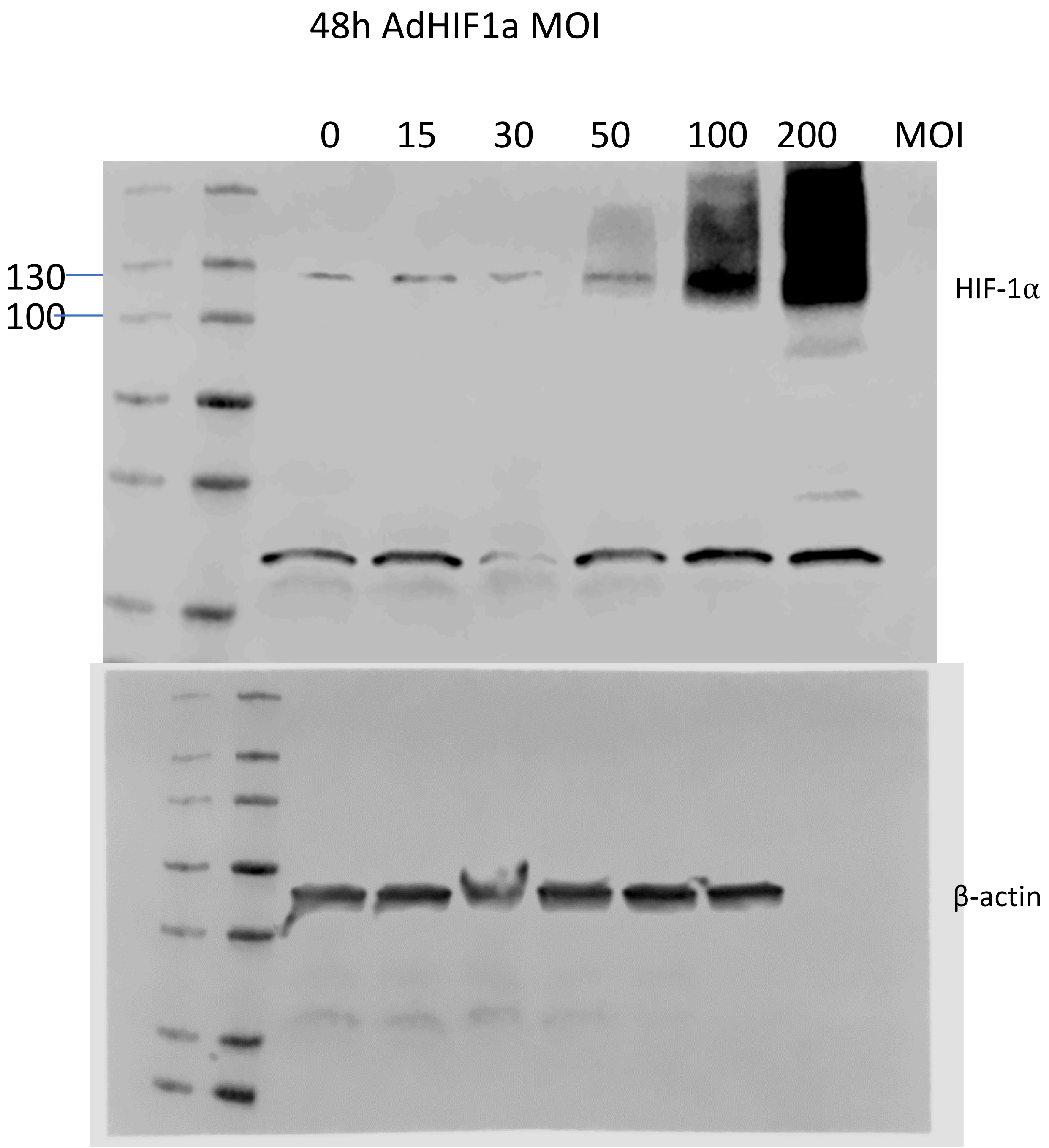

48h AdHIF1a MOI
0
15
30
50
100
200
MOI
130
100
HIF-1α
β-actin
